# Association of handgrip strength with new-onset CKD in Korean adults according to gender

**DOI:** 10.3389/fmed.2023.1148386

**Published:** 2023-06-20

**Authors:** Sung-Bum Lee, Miryung Kim, Hui-Jeong Lee, Jong-Koo Kim

**Affiliations:** ^1^Department of Family Medicine, Soonchunhyang University Bucheon Hospital, Bucheon, Republic of Korea; ^2^Department of Medicine, Graduate School, Yonsei University Wonju College of Medicine, Wonju, Republic of Korea; ^3^Department of Nephrology, Yonsei University Wonju College of Medicine, Wonju, Republic of Korea; ^4^Department of Family Medicine, Yonsei University Wonju College of Medicine, Wonju, Republic of Korea; ^5^Institute of Global Health Care and Development, Wonju, Republic of Korea

**Keywords:** handgrip strength, sarcopenia, chronic kidney disease, renal function, gender difference

## Abstract

**Introduction:**

Handgrip strength (HGS) is an indicator of many diseases such as pneumonia, cardiovascular disease and cancer. HGS can also predict renal function in chronic kidney disease (CKD) patients, but the value of HGS as a predictor of new-onset CKD is unknown.

**Methods:**

173,195 subjects were recruited from a nationwide cohort and were followed for 4.1  years. After exclusions, 35,757 participants remained in the final study, and CKD developed in 1063 individuals during the follow-up period. Lifestyle, anthropometric and laboratory data were evaluated in relation to the risk of CKD.

**Results:**

The participants were subdivided into quartiles according to relative handgrip strength (RGS). Multivariate Cox regression demonstrated that RGS was inversely associated with incident CKD. Compared with the lowest quartile, the hazard ratios (HRs) [95% confidence intervals (CIs)] for incident CKD for the highest quartile (Q4) was 0.55 (0.34–0.88) after adjusting for covariates in men and 0.51 (0.31–0.85) in women. The incidence of CKD decreased as RGS increased. These negative associations were more significant in men than in women. The receiver operating characteristic (ROC) curve showed that baseline RGS had predictive power for new-onset CKD. Area under the curve (AUC) (95% CIs) was 0.739 (0.707–0.770) in men and 0.765 (0.729–0.801) in women.

**Conclusion:**

This is the novel study demonstrating that RGS is associated with incident CKD in both men and women. The relationship between RGS and incident CKD is more significant in women than in men. RGS can be used in clinical practice to evaluate renal prognosis. Regular measurement of handgrip strength is essential to CKD detection.

## Introduction

CKD has been increasing over the past few decades. The all-stage mean global prevalence of CKD is 13.4% (1). Furthermore, CKD can aggravate hypertension, metabolic syndrome, and diabetes, all of which are risk factors for cardiovascular diseases (CVDs) (2, 3); and many studies have found associations of these comorbidities with CKD, including end-stage renal disease (ESRD) (4).

Prediction and early detection of CKD is an essential issue to prevent progression to ESRD, which can result in a range of complications, including malnutrition, anaemia, acidosis, and bone metabolism disorder (5). Nevertheless, individual willingness to prevent CKD acquisition and progression is often absent. Also, early CKD is often not diagnosed at an early stage due to the absence of clinical signs and symptoms. In the Third National Health and Nutrition Survey (NHANES III), only 8% of CKD patients understood the implications of their CKD diagnosis (6).

Sarcopenia, a comprehensive condition characterized by declining muscle strength and mass, is an essential public health concern worldwide. The global prevalence of sarcopenia was estimated at 10% (7). Sarcopenia is associated with several comorbidities such as pneumonia, falling, cardiovascular disease and cancer (8–10). For this reason, timely detection of sarcopenia is important. Handgrip strength is an inexpensive and convenient tool to evaluate muscle strength and is an effective method for diagnosing sarcopenia (11). Handgrip strength has been previously used to be a predictor of non-alcoholic fatty liver disease and healthy aging (12, 13). Recent studies have suggested that body mass index (BMI)-adjusted RGS is a more useful indicator than absolute handgrip strength (14). We, therefore, used RGS, absolute HGS divided by BMI, as the definitive measure in this study (14).

Several studies have already found a relationship between handgrip strength and CKD; however, these studies are cross-sectional studies (15, 16). In addition to investigating the association of RGS with CKD in Korean adults using nationwide cohort data, we assessed the utility of RGS as a predictor of new-onset CKD by using the data with the exclusion of baseline CKD.

## Materials and methods

### Study population

In the cohort study, data were collected from the Korean Genome and Epidemiology Study (KoGES) of the Korean general population. The KoGES data includes the KoGES_health examinee (HEXA) study, the KoGES_Ansan-Ansung study and the KoGES_cardiovascular disease association study (CAVAS). Our study used the KoGES HEXA study consisting of participants recruited from multiple clinics and aged ≥40 years at baseline. The population-based prospective cohort study was conducted to evaluate environmental and lifestyle determinants for the prevalence and incidence of chronic diseases (i.e., metabolic syndrome, obesity, hypertension, diabetes mellitus, osteoporosis, and CKD). Study design and information for KoGES have been previously described in detail (17).

**Figure 1:**
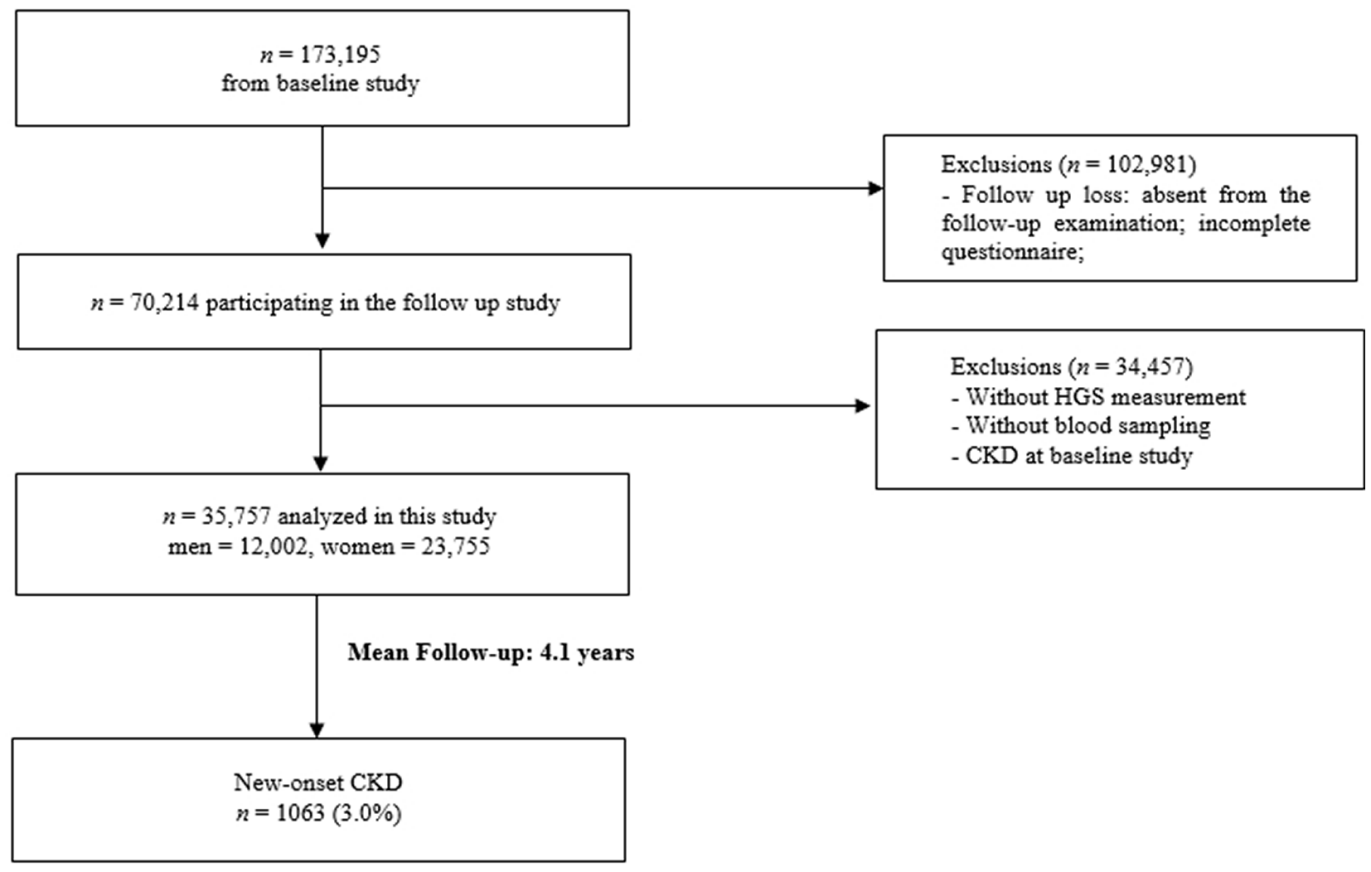
Flow diagram of studies meeting inclusion/exclusion criteria.

A total of 173,343 men and women aged 40–80 years participated in the baseline HEXA study conducted at 38 health centres nationwide from 2004 to 2013. A follow-up study was conducted from 2007 to 2016. Among the participants of the baseline study, we excluded those who: (1) were lost to follow-up, (2) were missing HGS data, (3) were missing laboratory data, or (4) were diagnosed with having CKD at the time of the baseline study (Figure 1).

The study protocol was approved by the Institutional Review Board (IRB) of the Yonsei University Wonju College of Medicine (IRB no. CR322322). This study was performed in compliance with the Declaration of Helsinki. Informed consent was acquired from all subjects, and all data were subsequently anonymized.

### Measurement of handgrip strength

Handgrip strength was measured twice with a 1 minute intervening rest interval using a digital grip strength dynamometer (T.K.K.5401, TAKEI Scientific Instruments Co., Ltd., Nigata, Japan) (18). The participants were trained to squeeze the dynamometer with as much force as possible. Each HGS was assessed after the grip was maintained at 15° from hip flexion. Absolute HGS was defined as the maximum value from both hands and was presented in kilograms (14). In order to normalize the impact of body size on HGS, relative handgrip strength (RGS) was used. RGS was defined as the absolute HGS divided by BMI, which had been previously used as indicator for muscle strength (14). The RGS data were subdivided into gender-specific quartiles.

### Anthropometric and laboratory measurements and general data

Anthropometric, demographic, lifestyle, and laboratory data were collected from all the participants. The anthropometric data included gender, age, waist circumference (WC), BMI, systolic blood pressure (SBP), and diastolic blood pressure (DBP). WC was measured using flexible tape (Seca 220; Seca) at the midpoint between the lowest margin of the rib and the uppermost border of the iliac crest during expiration (19). The BMI was calculated as weight divided by height squared (kg/m^2^). Blood pressure (BP) was measured using a mercury sphygmomanometer after the subjects rested for five min in a sitting position (Baumanometer Wall Unit 33(0850)). All BP examinations were performed on the right arm twice using the same tool at 30 s intervals (20). Hypertension was diagnosed as SBP ≥ 140 mmHg, DBP ≥ 90 mmHg or being administered antihypertensive drugs (21). Diabetes mellitus was defined as when one of the following criteria based on the American Diabetes Association (ADA) criteria was met: fasting plasma glucose ≥126 mg/dL, HbA1c ≥ 6.5%, or plasma glucose level 2 h after 75 g OGTT ≥ 200 mg/dL (22). Participants who answered “yes” to taking diabetes medication were also regarded as diabetes. The medication history was collected using questionnaires. In addition to the medication history, the participants answered the questionnaires including information on demographics, lifestyle, and medical conditions: gender, age, alcohol intake, smoking history, regular exercise, and current and past medical history of diseases. Alcohol history data were collected using questionnaires including the type (beer, hard liquor, and soju), amount, and frequency of drinks. Alcohol intake was defined as drinking at least once a week; the cut-off for amount of alcohol intake per week was >140 g for men and >70 g for women (23). Smoking history was categorized as current smokers, ex-smokers, and never smokers. Current smokers were regarded as those who responded “yes” to the statement “I have smoked more than 5 packs of cigarettes in a lifetime and still smoke.” Ex-smokers were regarded as those who answered “yes” to the statement “I have smoked more than 5 packs of cigarettes but do not smoke anymore.” Never smokers were regarded as those who answered “yes” to the statement “I have smoked less than 5 packs of cigarettes in a lifetime” (24). Regular exercise was defined as engaging in vigorous physical activity more than 3 times per week. Patients with cardiovascular disease were considered as those who responded “yes” to the statement “I have been diagnosed with cardiovascular disease by a physician.” The global physical activity questionnaire (GPAQ) was applied to assess the level of physical activity (25). Aspartate aminotransferase (AST), alanine aminotransferase (ALT), total cholesterol (TC), low-density lipoprotein (LDL) cholesterol and c-reactive protein (CRP) levels were measured by high-performance liquid chromatography with an automated HGLC-723G7 analyser (Tosoh Corporation, Tokyo, Japan).

### Definition of chronic kidney disease

CKD was defined as an estimated glomerular filtration rate (eGFR) less than 60 mL/min/1.73 m^2^ or proteinuria ≥1+ according to the Kidney Disease Outcomes Quality Initiative (KDOQI) CKD classification (26). eGFR was calculated using the equation from the chronic kidney disease epidemiology collaboration, CKD-EPI 2021. This equation is (27):


$$\begin{array}{l} \mathrm{eGFR}=142\times \min{\left(\frac{S_{\mathrm{cr}}}{K},1\right)}^{\alpha }\times \max{\left(\frac{S_{\mathrm{Cr}}}{K},1\right)}^{-1.200}\times 0.9938^{\mathrm{Age}}\\ \times 1.012\;\left[\mathrm{if female}\right] \end{array}$$

S_cr_ (Serum creatinine) = mg/dL; *K* = 0.7 (females) or 0.9 (males); *α* = −0.241 (females) or −0.302 (males); Min = indicates the minimum of S_cr_ or 1; Max = indicates the minimum of S_cr_ or 1.

Participants who reported being diagnosed with CKD by physicians were also regarded as having CKD.

### Statistical analysis

All covariates were analysed by chi-square test for categorical variables and independent *t*-test and analysis of variance (ANOVA) tests for continuous variables. The categorical and continuous variables were expressed as *n* (%) and mean ± standard deviation, respectively (Table 1). Cox regression analysis was conducted to evaluate the association of RGS (per 0.01 kg) with incidence of CKD after adjusting for age; alcohol consumption; smoking status; regular exercise; and SBP, DBP, AST, ALT, TC, LDL-cholesterol, and CRP levels (Table 2). RGS data were subdivided into quartiles: Q1, ≤1.36; Q2, 1.36–1.57; Q3, 1.57–1.79; and Q4, >1.79 in men. For women, these values were Q1 ≤ 0.84; Q2, 0.84–1.00; Q3, 1.00–1.16; and Q4, >1.16. The weakest RGS group (Q1) was defined as the reference group. Cox regression was performed to calculate the HRs and 95% CIs of incident CKD for RGS quartiles after adjusting for the confounding factors (Table 3). ROC curves were illustrated to analyse the predictive power for new-onset CKD according to baseline RGS, and AUC was calculated. Kaplan–Meier curves were constructed to assess survival probability for incident CKD according to baseline RGS quartiles. Value of *p* <0.05 was considered statistically significant. Statistical analyses were conducted using SPSS version 27.0 (IBM Corp., Armonk, NY, United States).

**Table 1:** Baseline characteristics of study population according to baseline RGS quartile.

	Men	Q_1_	Q_2_	Q_3_	Q_4_	value of *p*
		≤1.38	1.38–1.59	1.59–1.82	>1.82	
*N*	12,002	2,983	2,962	3,074	2,983	
HGS (kg)	38.9 ± 8.4	29.9 ± 6.0	37.0 ± 3.7	41.1 ± 4.3	47.3 ± 7.6	<0.001
RGS (m^2^)	1.61 ± 0.37	1.16 ± 0.20	1.49 ± 0.06	1.70 ± 0.07	2.06 ± 0.26	<0.001
Age (years)	55.2 ± 8.4	58.3 ± 8.0	56.4 ± 8.0	54.7 ± 7.9	51.3 ± 7.9	<0.001
Waist circumference (cm)	85.4 ± 7.5	88.5 ± 7.5	86.5 ± 6.8	84.9 ± 7.0	81.7 ± 7.0	<0.001
BMI (kg/m^2^)	24.4 ± 2.7	25.7 ± 2.8	24.9 ± 2.4	24.2 ± 2.4	22.9 ± 2.4	<0.001
eGFR (mL/min/1.73 m^2^)	94.5 ± 11.8	92.7 ± 11.7	93.6 ± 11.7	94.8 ± 11.8	96.9 ± 11.7	<0.001
Total cholesterol (mg/dl) (mg/dl)	191.9 ± 34.8	191.3 ± 36.2	192.6 ± 35.5	192.8 ± 34.4	191.0 ± 33.0	0.110
LDL-cholesterol (mg/dl)	113.8 ± 31.3	113.6 ± 32.4	114.4 ± 32.2	114.0 ± 30.9	113.3 ± 29.5	0.494
HDL-cholesterol (mg/dl)	49.7 ± 11.9	48.1 ± 11.2	48.9 ± 11.8	50.0 ± 11.8	51.7 ± 12.5	<0.001
Triglyceride (mg/dl)	148.6 ± 102.7	154.4 ± 101.3	152.8 ± 102.0	150.8 ± 106.4	136.2 ± 100.0	<0.001
Albumin (mg/dl)	4.69 ± 0.25	4.67 ± 0.26	4.69 ± 0.26	4.70 ± 0.25	4.72 ± 0.25	<0.001
AST (IU/L)	25.0 ± 13.0	25.5 ± 11.8	25.5 ± 14.5 25.5 ± 14.4	25.1 ± 13.7	24.0 ± 11.6	<0.001
ALT (IU/L)	25.9 ± 16.9	27.5 ± 17.0	26.8 ± 17.1	25.9 ± 17.4	23.4 ± 15.5	<0.001
CRP (mg/dL)	0.159 ± 0.389	0.185 ± 0.430	0.163 ± 0.361	0.144 ± 0.308	0.140 ± 0.443	<0.001
Systolic BP (mmHg)	125.5 ± 13.9	127.2 ± 14.2	125.9 ± 13.6	125.4 ± 13.7	123.7 ± 13.7	<0.001
Diastolic BP (mmHg)	78.1 ± 9.4	78.9 ± 9.4	78.2 ± 9.2	77.8 ± 9.3	77.2 ± 9.5	<0.001
Alcohol intake, *n* (%)	4,109 (34.3)	905 (30.4)	966 (32.6)	1,125 (36.6)	1,113 (37.3)	<0.001
Smoking status, *n* (%)						<0.001
Never smoker	3,190 (26.7)	863 (29.1)	770 (26.1)	806 (26.3)	751 (25.3)	
Ex-smoker	5,452 (45.6)	1,429 (48.1)	1,374 (46.5)	1,407 (46.0)	1,242 (41.8)	
Current smoker	3,316 (27.7)	678 (22.8)	811 (27.4)	846 (27.7)	981 (33.0)	
Regular exercise, *n* (%)	5,083 (42.6)	1,284 (43.3)	1,301 (44.1)	1,325 (43.4)	1,173 (39.5)	0.001
Hypertension, *n* (%)	2,952 (24.6)	1,027 (34.5)	824 (27.8)	690 (22.5)	411 (13.8)	<0.001
Diabetes, *n* (%)	1,138 (9.5)	404 (13.6)	329 (11.1)	262 (8.5)	143 (4.8)	<0.001
CVD, *n* (%)	522 (4.3)	176 (5.9)	151 (5.1)	123 (4.0)	72 (2.4)	<0.001

	Women	Q_1_	Q_2_	Q_3_	Q_4_	value of *p*
		≤0.84	0.84–1.00	1.00–1.16	>1.16	
*N*	23,755	5,828	6,059	5,927	5,941	
HGS (kg)	23.4 ± 5.3	17.8 ± 3.7	22.3 ± 2.5	24.9 ± 2.6	28.6 ± 4.6	<0.001
RGS (m^2^)	1.01 ± 0.25	0.70 ± 0.13	0.92 ± 0.05	1.08 ± 0.05	1.32 ± 0.18	<0.001
Age (years)	53.3 ± 7.8	56.9 ± 7.5	54.4 ± 7.4	52.3 ± 7.2	49.5 ± 6.9	<0.001
Waist circumference (cm)	77.9 ± 8.1	82.2 ± 8.4	79.3 ± 7.6	76.8 ± 7.1	73.4 ± 6.7	<0.001
BMI (kg/m^2^)	23.5 ± 2.9	25.4 ± 3.2	24.2 ± 2.6	23.1 ± 2.3	21.7 ± 2.2	<0.001
eGFR (mL/min/1.73 m^2^)	99.2 ± 11.2	97.4 ± 11.1	99.0 ± 11.0	99.8 ± 11.1	100.7 ± 11.2	<0.001
Total cholesterol (mg/dl) (mg/dl)	199.8 ± 35.5	202.4 ± 36.9	202.0 ± 35.9	199.9 ± 35.2	195.0 ± 33.7	<0.001
LDL-cholesterol (mg/dl)	121.2 ± 32.0	123.7 ± 33.3	123.2 ± 32.4	121.3 ± 31.5	116.4 ± 30.0	<0.001
HDL-cholesterol (mg/dl)	56.3 ± 13.0	53.8 ± 12.2	55.2 ± 12.6	56.8 ± 12.9	59.5 ± 13.5	<0.001
Triglyceride (mg/dl)	113.6 ± 72.6	127.0 ± 76.1	120.6 ± 79.5	110.4 ± 68.7	96.5 ± 60.7	<0.001
Albumin (mg/dl)	4.61 ± 0.24	4.58 ± 0.25	4.61 ± 0.25	4.62 ± 0.24	4.64 ± 0.24	<0.001
AST (IU/L)	22.2 ± 10.3	23.4 ± 11.2	22.7 ± 12.8	21.8 ± 9.0	20.9 ± 7.2	<0.001
ALT (IU/L)	19.6 ± 15.1	22.0 ± 18.2	20.4 ± 17.7	18.8 ± 11.7	17.1 ± 10.8	<0.001
CRP (mg/dL)	0.131 ± 0.396	0.165 ± 0.410	0.146 ± 0.516	0.121 ± 0.333	0.091 ± 0.270	<0.001
SBP (mmHg)	120.9 ± 14.7	123.2 ± 14.7	121.9 ± 14.8	120.6 ± 14.8	118.0 ± 14.1	<0.001
DBP (mmHg)	74.2 ± 9.4	75.4 ± 9.3	74.7 ± 9.3	74.1 ± 9.4	72.7 ± 9.3	<0.001
Alcohol intake, *n* (%)	1,114 (4.7)	202 (3.5)	265 (4.4)	303 (5.1)	344 (5.8)	<0.001
Smoking status, *n* (%)						0.017
Never smoker	22,890 (96.9)	5,630 (97.1)	5,840 (97.0)	5,726 (97.2)	5,694 (96.2)	
Ex-smoker	295 (1.2)	73 (1.3)	65 (1.1)	74 (1.3)	83 (1.4)	
Current smoker	439 (1.9)	94 (1.6)	115 (1.9)	91 (1.5)	139 (2.3)	
Regular exercise, *n* (%)	9,955 (42.1)	2,255 (38.9)	2,524 (41.9)	2,615 (44.4)	256 (43.3)	<0.001
Hypertension, *n* (%)	4,215 (17.8)	1,553 (26.7)	1,204 (19.9)	903 (15.3)	555 (9.3)	<0.001
Diabetes, *n* (%)	1,226 (5.2)	521 (8.9)	341 (5.6)	227 (3.8)	137 (2.3)	<0.001
CVD, *n* (%)	518 (2.2)	237 (4.1)	143 (2.4)	96 (1.6)	42 (0.7)	<0.001

HGS, handgrip strength; RGS, relative handgrip strength; BMI, body mass index; eGFR, estimated glomerular filtration rate; LDL, low density lipoprotein; HDL, high density lipoprotein; AST, aspartate aminotransferase; ALT, alanine aminotransferase; CRP, c-reactive protein; SBP, systolic blood pressure; DBP, diastolic blood pressure; CVD, cardiovascular disease.

**Table 2:** Association between baseline RGS (per 0.01 kg) and incidence of CKD in Koreans using cox-regression.

Men			Women		
	HR	value of *p*		HR	value of *p*
Unadjusted	0.46 (0.35–0.60)	<0.001	Unadjusted	0.68 (0.49–0.94)	0.018
Model 1	0.60 (0.45–0.80)	0.001	Model 1	0.67 (0.48–0.94)	0.022
Model 2	0.60 (0.45–0.80)	0.001	Model 2	0.66 (0.48–0.92)	0.012
Model 3	0.68 (0.48–0.98)	0.037	Model 3	0.80 (0.51–1.26)	0.331

CKD, chronic kidney disease; HR, hazard ratio; Model 1: adjusted for age; Model 2: adjusted for age, regular exercise, alcohol intake, and smoking status; Model 3: adjusted for age, hypertension, diabetes, cardiovascular disease, regular exercise, alcohol intake, smoking status, BMI, SBP, DBP, AST, ALT, albumin, proteinuria, eGFR, total cholesterol, HDL-cholesterol, triglyceride, and CRP.

**Table 3:** Hazard ratio and 95% confidence intervals for new-onset CKD according to baseline RGS quartile.

	Men				Women			
	Q_1_	Q_2_	Q_3_	Q_4_	Q_1_	Q_2_	Q_3_	Q_4_
	≤1.36	1.36–1.57	1.57–1.79	>1.79	≤0.84	0.84–1.00	1.00–1.16	>1.16
*n*	2,961	2,947	3,050	2,970	5,828	6,059	5,927	5,941
Unadjusted	1.00	0.68 (0.53–0.87)	0.67 (0.53–0.86)	0.39 (0.29–0.53)	1.00	0.93 (0.75–1.14)	0.85 (0.68–1.06)	0.78 (0.62–0.97)
Model 1	1.00	0.73 (0.57–0.94)	0.78 (0.61–1.00)	0.51 (0.37–0.70)	1.00	0.92 (0.75–1.14)	0.85 (0.68–1.06)	0.77 (0.61–0.98)
Model 2	1.00	0.76 (0.59–0.97)	0.83 (0.64–1.06)	0.57 (0.42–0.78)	1.00	0.95 (0.77–1.18)	0.89 (0.71–1.12)	0.86 (0.68–1.10)
Model 3	1.00	0.62 (0.46–0.85)	0.83 (0.62–1.13)	0.55 (0.37–0.82)	1.00	1.06 (0.82–1.38)	0.99 (0.75–1.32)	0.84 (0.60–1.16)

Model 1: adjusted for age; Model 2: adjusted for age, regular exercise, alcohol intake, and smoking status; Model 3: adjusted for age, hypertension, diabetes, cardiovascular disease, regular exercise, alcohol intake, smoking status, BMI, SBP, DBP, AST, ALT, albumin, proteinuria, eGFR, total cholesterol, HDL-cholesterol, triglyceride, and CRP.

## Results

Baseline characteristics of the study population according to baseline RGS quartile are described in Table 1. A total of 35,757 participants (12,002 men, 23,755 women) were included in our study. The mean values of some covariates were significantly decreased with increasing RGS quartile. These variables were age, WC, BMI, AST, ALT, CRP, SBP, DBP, presence of hypertension, and presence of diabetes in men. In women, these variables were age, WC, BMI, TC, LDL-cholesterol, AST, ALT, CRP, SBP, DBP, presence of hypertension, and presence of diabetes.

**Figure 2:**
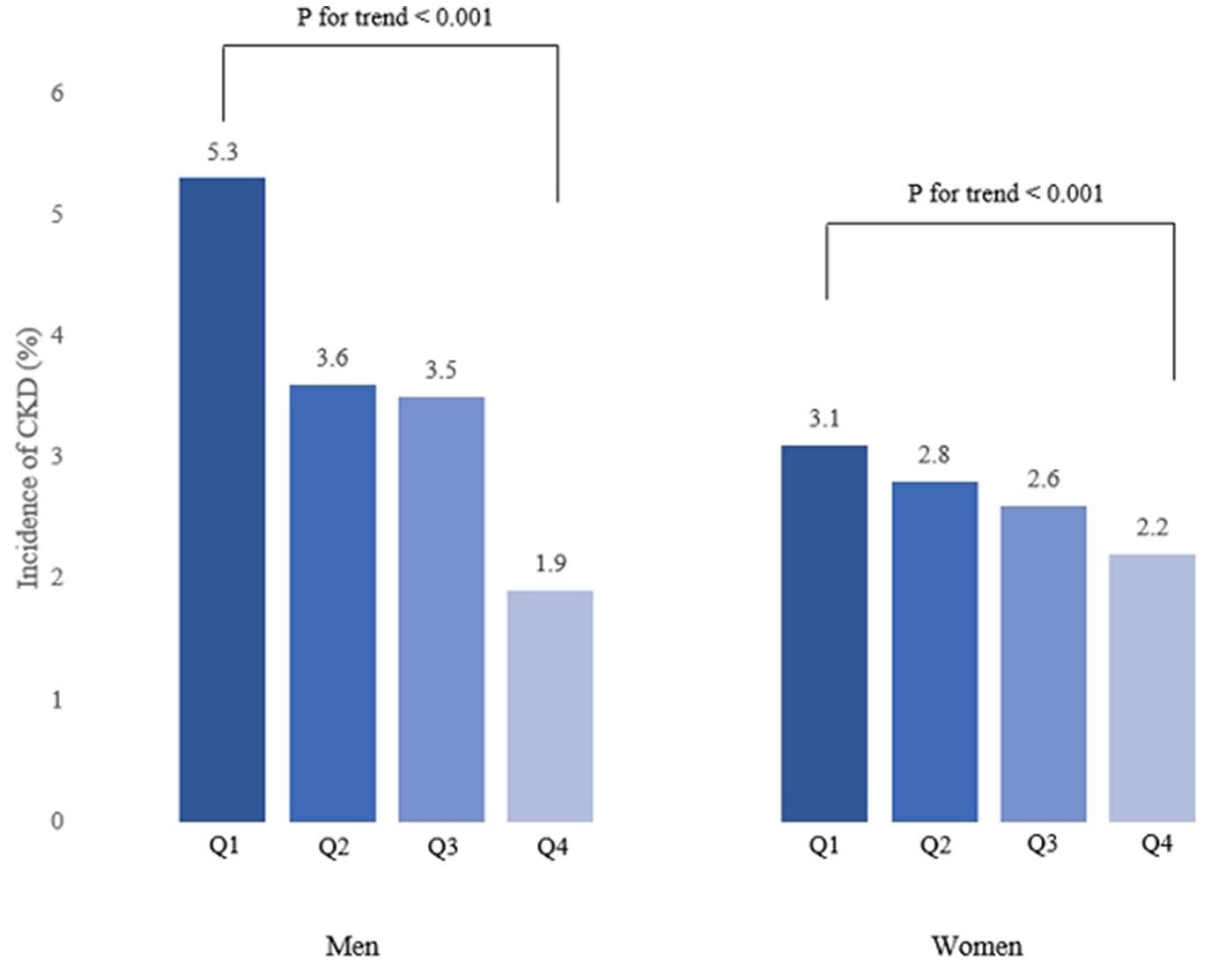
Incidence of CKD according to baseline RGS quartiles.

The incidence of CKD decreased with increasing baseline RGS quartile in both men and women (Figure 2). These results suggest that dose–response relationship was present between RGS and CKD. The results of the relationship between baseline RGS (per 0.01 kg) and incidence of CKD in Koreans are tabulated in Table 2. RGS was inversely related to incidence of CKD in all models for both men and women.

Table 3 shows the HRs and 95% CIs for the incidence of CKD according to baseline RGS quartile. The weakest quartile (Q1) of RGS was defined as the reference group (14). Compared with the reference group and after adjusting model 3, the statistically significant HRs (95% CI) for CKD of the participants were 0.55 (0.34–0.88) for the Q4 group of men, 0.43 (0.27–0.69) for the Q3 group of women, and 0.51 (0.31–0.85) for the Q4 group of women.

**Figure 3:**
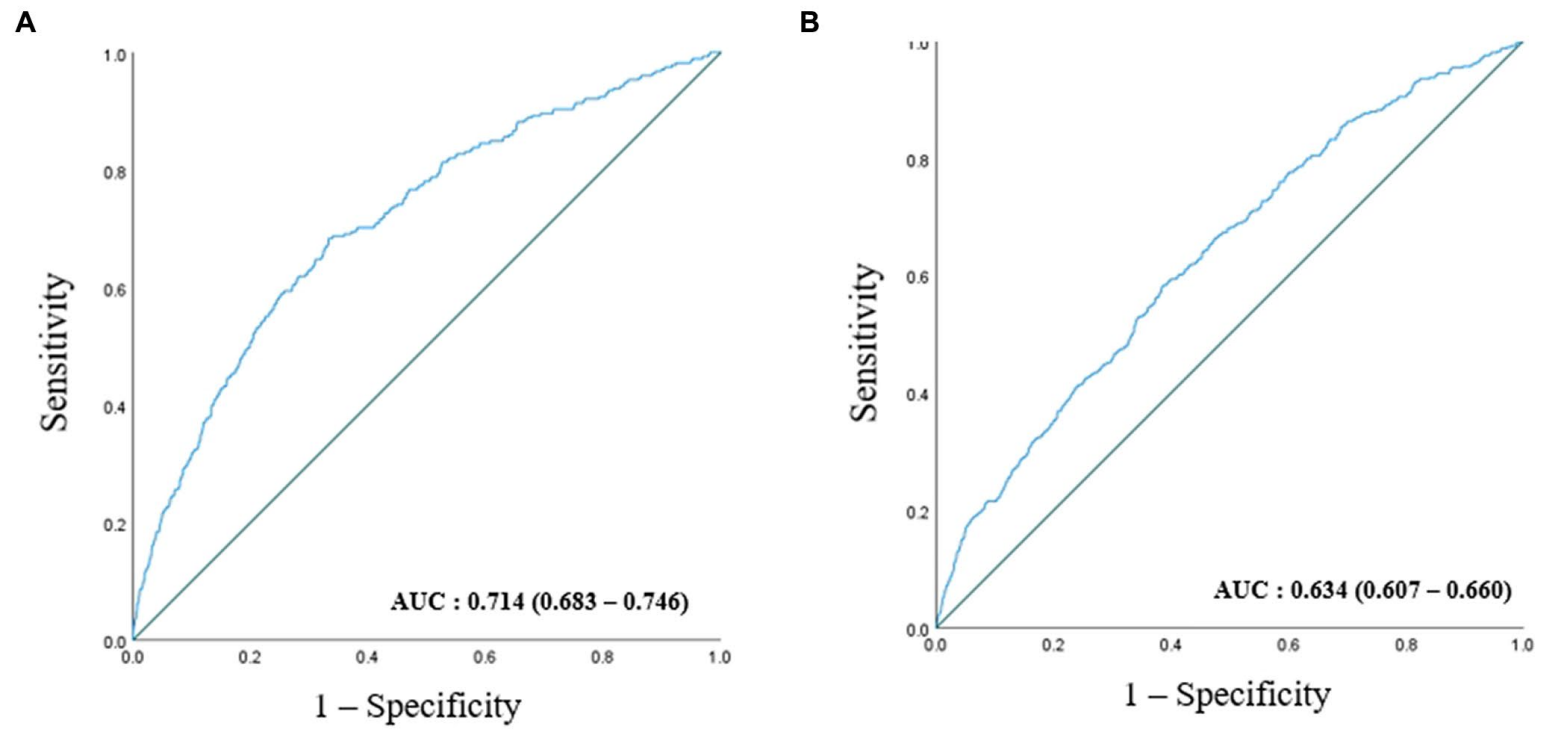
ROC curve presenting the predictive power for incident CKD according to baseline RGS in men **(A)** and in women **(B)**.

**Figure 4:**
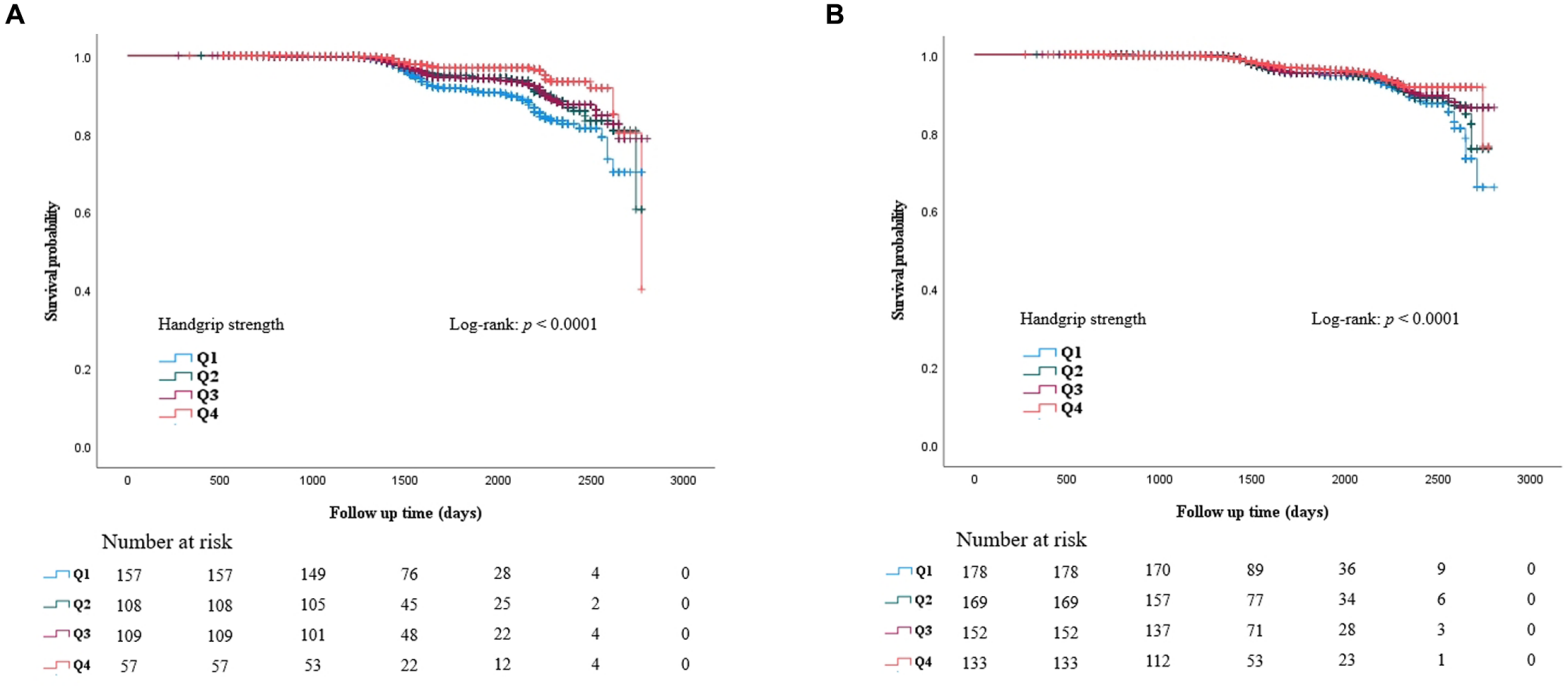
Kaplan Meier curve for incident CKD according to baseline RGS quartile in men **(A)** and in women **(B)**.

ROC curves were generated to test RGS as a predictor of CKD (Figure 3). The AUC of Figure 3A is 0.739 (0.707–0.770), and the AUC of Figure 3B is 0.765 (0.729–0.801). During the 90 months of follow-up, new-onset CKD developed in 1063 individuals (3.0%, 1063/35,757). The survival rates without incident CKD were lowest in the Q2 group up to 90 months but increased gradually from Q2 to Q4 in both men and women after the baseline survey (log-rank test, *p* < 0.001) (Figure 4).

## Discussion

In the nationwide cohort study conducted over 12 years, RGS was inversely associated with the incidence of CKD. Furthermore, relative handgrip strength was an independent predictor of CKD, irrespective of age; regular exercise; alcohol intake; smoking history; and SBP, DBP, AST, ALT, TC, LDL-cholesterol, and CRP levels.

Handgrip strength is a prognostic indicator for metabolic syndrome, hypertension and diabetes (28–30). However, these studies commonly suggested that HGS be a practical tool to evaluate these comorbidities in high-risk groups rather than healthy groups. Moreover, several studies have found an association between handgrip strength and CKD (31, 32). However, these studies only demonstrated that HGS is an independent predictor of renal function in CKD patients. The findings of our study are the novel to identify that RGS is a useful tool to predict new-onset CKD. We demonstrated this by excluding subjects with CKD at baseline and by having a large subject sample size. The association remained significant after adjusting for age; blood pressure; and TC, LDL-cholesterol and CRP levels, all of which are risk factors for CKD.

Measurement of handgrip strength is a practical tool to evaluate muscle strength because of its low cost and ease of implementation (33). Low muscle strength, not low muscle mass, is a primary determinant of sarcopenia; muscle strength is a more reliable predictive indicator of falling, fracture, and all-cause mortality than muscle mass (9, 34). Accordingly, handgrip strength is commonly used as a diagnostic approach for sarcopenia.

Even though there are many mechanisms of sarcopenia in CKD patients (35), a mechanical link between sarcopenia and incident CKD has not been fully elucidated. Therefore, we propose putative mechanisms involving mediators of sarcopenia and CKD, e.g., oxidative stress, inflammation, and insulin resistance (IR) (36, 37). Skeletal muscles have an important role in glucose homeostasis; skeletal muscle accounts for 40–50% of lean body mass in an adult and, therefore, is the source of most of the body’s insulin-stimulated glucose disposal (38). Sarcopenia can lead to the inevitable deterioration of skeletal muscle cell structure and biological function (39) and can impair insulin-stimulated glucose disposal into muscle thereby impacting glucose homeostasis (38). Several sarcopenia-associated features such as mitochondrial dysfunction, increased inflammation, and increased oxidative stress arise; these factors cause IR (40). Decreased muscle strength can involve changes in released inflammatory markers. Several studies have shown that lower HGS is associated with higher levels of inflammatory markers such as interleukin (IL)-6 and tumour necrosis factor (TNF)-alpha. The inflammatory markers affect the maintenance of metabolic homeostasis (41, 42). Through the proposed mechanisms, several studies have previously reported that handgrip strength can be a predictor for the incidence of metabolic syndrome and diabetes (18, 43).

Insulin plays an essential role in glucose metabolism, and the kidney is an insulin target organ because the kidney plays an important role in the clearance and degradation of insulin (44). If cells, particularly kidney cells, fail to respond to insulin, the resulting IR can lead to CKD. Moreover, IR can promote the development of atherosclerosis, hypertension, dyslipidaemia, fatty liver, and obesity, all of which are important risk factors for CKD (13, 45–48).

In spite of many advantages, there are several limitations to our study. First, decreased eGFR should be maintained for at least 3 months according to the KDOQI definition of CKD (49). However, eGFR less than 60 at the first follow-up was the diagnostic criterion in our study. Because the KoGES HEXA study was conducted in multiple clinics with the recruitment of large number of participants, the maintenance of decreased eGFR was difficult to assess through short term follow-up. Several previous studies also defined CKD as eGFR less than 60. In one study this was true regardless of the maintenance of decreased eGFR (50, 51). Second, although RGS adjusted for BMI was used, our study could not reflect muscle mass because there are no muscle mass data in the KoGES. Therefore, we could not ascertain that the relationship between handgrip strength and CKD was independent of muscle mass. Nonetheless, handgrip strength was used in our study because previous studies determined this to be a more useful tool than muscle mass (34). Sarcopenia is regarded as muscle failure with low muscle strength being a superior measure to a lack of muscle mass (52, 53). Third, the event date of CKD may have been different from the follow-up date. Because the follow-up cohort study had been conducted regardless of the occurrence of CKD, the occurrence date was not always the same date as the follow-up date. Furthermore, participants who might die during follow-up could not be included in our study because they were excluded due to follow-up loss. KoGES did not have mortality data. Fourth, dipstick test is not a quantitative test such as 24 h urine collection. Therefore, it can miss albuminuria. Finally, a proper index to eliminate the effect of body size (weight, height, and BMI) on handgrip strength has not yet been established. Even though RGS can minimize the impact of body size, dividing HGS by BMI cannot completely correct for this effect (54). Nevertheless, RGS has been widely used for lessening body size effects (14). Further studies are needed for muscle strength-associated indices independent of body size.

## Conclusion

We found that RGS was independently negatively associated with new-onset CKD in men and women. The association of handgrip strength with incident CKD is more significant in men than in women. RGS can be a useful tool to predict the incidence of CKD. The appropriate measurement of handgrip strength is important to detect CKD.

## Data availability statement

The original contributions presented in the study are included in the article/supplementary material, further inquiries can be directed to the corresponding author.

## Ethics statement

The studies involving human participants were reviewed and approved by Institutional Review Board (IRB) of the Yonsei University Wonju College of Medicine. The patients/participants provided their written informed consent to participate in this study.

## Author contributions

J-KK: conceptualization and writing-editing. H-JL and S-BL: methodology. S-BL: formal analysis. S-BL and J-KK: investigation. MK and S-BL: writing-original draft preparation. H-JL and J-KK: supervision. All authors have read and agreed to the published version of the manuscript. All authors contributed to the article and approved the submitted version.

## Conflict of interest

The authors declare that the research was conducted in the absence of any commercial or financial relationships that could be construed as a potential conflict of interest.

## Publisher’s note

All claims expressed in this article are solely those of the authors and do not necessarily represent those of their affiliated organizations, or those of the publisher, the editors and the reviewers. Any product that may be evaluated in this article, or claim that may be made by its manufacturer, is not guaranteed or endorsed by the publisher.
